# Web-Based Computational Chemistry Education with CHARMMing I: Lessons and Tutorial

**DOI:** 10.1371/journal.pcbi.1003719

**Published:** 2014-07-24

**Authors:** Benjamin T. Miller, Rishi P. Singh, Vinushka Schalk, Yuri Pevzner, Jingjun Sun, Carrie S. Miller, Stefan Boresch, Toshiko Ichiye, Bernard R. Brooks, H. Lee Woodcock

**Affiliations:** 1 Laboratory of Computational Biology, National Heart, Lung, and Blood Institute, Bethesda, Maryland, United States of America; 2 Department of Natural Sciences, New College of Florida, Sarasota, Florida, United States of America; 3 Department of Chemistry, University of South Florida, Tampa, Florida, United States of America; 4 Department of Chemistry, Georgetown University, Washington, D.C., United States of America; 5 Department of Computational Biological Chemistry, Faculty of Chemistry, University of Vienna, Vienna, Austria; University of Wisconsin-Madison, United States of America

## Abstract

This article describes the development, implementation, and use of web-based “lessons” to introduce students and other newcomers to computer simulations of biological macromolecules. These lessons, i.e., interactive step-by-step instructions for performing common molecular simulation tasks, are integrated into the collaboratively developed CHARMM INterface and Graphics (CHARMMing) web user interface (http://www.charmming.org). Several lessons have already been developed with new ones easily added via a provided Python script. In addition to CHARMMing's new lessons functionality, web-based graphical capabilities have been overhauled and are fully compatible with modern mobile web browsers (e.g., phones and tablets), allowing easy integration of these advanced simulation techniques into coursework. Finally, one of the primary objections to web-based systems like CHARMMing has been that “point and click” simulation set-up does little to teach the user about the underlying physics, biology, and computational methods being applied. In response to this criticism, we have developed a freely available tutorial to bridge the gap between graphical simulation setup and the technical knowledge necessary to perform simulations without user interface assistance.

## Introduction

An accurate model of a complex molecular system can provide a great deal of insight into its properties and behavior; however, obtaining this information from simulation studies can be a daunting task. For example, the practitioner is required to possess in-depth knowledge of three key aspects: the system of interest, techniques to be applied, and the technical skills to employ the necessary software packages. As has been previously described, simulations are also useful for allowing students to explore basic concepts [1], [2]. However, common molecular simulation packages can be intimidating for students and other novice users. This is true of Chemistry at HARvard Macromolecular Mechanics (CHARMM) [3], one of the most widely used and feature-rich simulation programs. Another factor limiting the use of simulations for educational purposes is that certain methodologies such as molecular dynamics (MD) require a large quantity of dedicated computational resources. Furthermore, such calculations may require a complex series of setup runs that can be difficult for students to perform. To overcome this, CHARMMing (CHARMM interface and graphics, available at http://www.charmming.org) [4] has been developed to provide a user-friendly “sandbox” that allows users to learn simulation techniques rather than the details of a particular software package. In addition to CHARMMing, numerous other web-based interfaces to modeling and simulations packages have been developed; however, most of the existing web interfaces have a very narrow focus (e.g., input generation for particular calculations) and do not emphasize educational use as a specific goal [5]–[10].

In contrast to highly specialized tools, CHARMMing is well suited for adaptation to an education-centric framework. For example, a user can upload a structure and perform a variety of operations, including minimization, solvation, dynamics, and more in any order. Furthermore, the user is not limited to one particular type of calculation with all-atom classical, coarse-grained, and even quantum mechanical methods supported. This flexibility offers limitless possibilites for developing basic, intermediate, and advanced level workflows that are currently lacking. To this end, the current paper describes three key developments that greatly enhance CHARMMing as a pedagogical aid:

1. Interactive **lessons** designed to guide novices through step-by-step directions for performing common molecular simulation tasks.
2. Enhanced **visualization tools** that are used to graphically set up complex calculations, allow richer display of structures and dynamics, and extend the applicibility of CHARMMing to a mobile computing environment (e.g., phones, tablets).
3. A **tutorial** designed to bridge the gap between graphical simulation setup and the technical knowledge necessary to perform simulations without user interface assistance. In particular, working through the tutorial and performing sample calculations allows students to understand and modify the CHARMM scripts that CHARMMing produces.

As described by Paniagua et al. [11], the problem of software complexity can largely be solved with either (i) “friendly web interface(s)” or (ii) by distributing bootable CDs with preinstalled software. The addition of lessons to CHARMMing follows strategy (i) and exploits CHARMMing's web interface coupled with a powerful simulation package, CHARMM. Furthermore, a tutorial has been created that explains some of the physics behind how CHARMM performs simulations and the program's command line syntax. This allows novices who have some background in the methods being employed (vide infra) to bridge the gap between the web interface and independent CHARMM usage.

The purpose of CHARMMing's lessons and tutorial is not to teach from scratch the concepts behind molecular mechanics and simulation. It is assumed that the user has some knowledge of classical and, to a much lesser extent, quantum mechanics. In particular, he or she should have some background in the structure of proteins and other biopolymers and a basic understanding of how their potential energy and movements can be calculated using classical mechanics. Knowledge of sources of structural data (e.g., X-ray crystallography, Nuclear Magnetic Resonance spectroscopy, etc.) and a basic understanding of statistical mechanics (e.g., partition functions, various ensembles) will enhance understanding of the material but are not strictly required. This information can come from a variety of sources; e.g., classroom instruction or textbooks [12]–[16].

## Implementation of Lessons into CHARMMing

### 

CHARMMing is an open source, web user interface for the CHARMM simulation package. Although CHARMMing can be downloaded and installed locally, it still requires a CHARMM license for full functionality, which includes energy evaluation, minimization, dynamics, normal mode analysis, and more. In order to bridge the gap between the web interface and tutorial-style introductions to the underlying software, interactive lessons have been added to CHARMMing. In this way, students may learn to use CHARMMing to perform techniques such as MD, mixed quantum–classical calculations, trajectory visualization, Oxidation/Reduction calculations, and more. Key design considerations for the lessons are as follows:

#### 1. Provide real-time progress tracking

Students cannot be initially expected to understand the “bigger picture” of what is involved with setting up and running a simulation or to interpret complex output from a program like CHARMM. While documentation exists, a novice user may not be able to clearly relate their own outputs to it and to results reported in the literature. Therefore, the lessons system must clearly show each step in a calculation and whether or not it has been performed successfully.

#### 2. Introduce the user to different types of simulations

Since molecular modeling is a broad field, the infrastructure should limit the lesson implementer as little as possible. Reasonable default values are given along with step-by-step instructions for performing various tasks.

#### 3. Make it easy to implement additional lessons

Anyone with some knowledge of the Python programming language should be able to implement their own lesson without having to learn the entirety of the CHARMMing source code. CHARMMing accomplishes this by providing “hooks” at various points in the workflow. The only requirement, then, is to write the particular logic needed for each step and the accompanying user-facing instructions.

**Figure 1:**
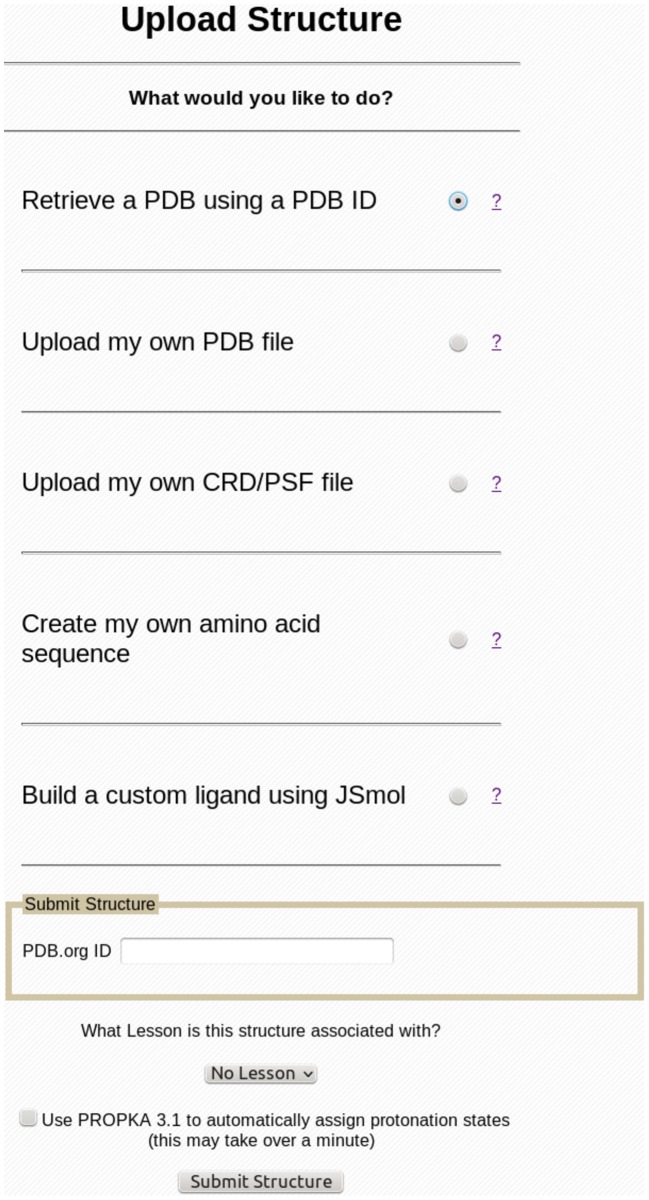
The file upload page of CHARMMing version 0.10. A drop-down menu allowing the user to select a lesson that the new structure is to be used for has been added.

**Figure 2:**
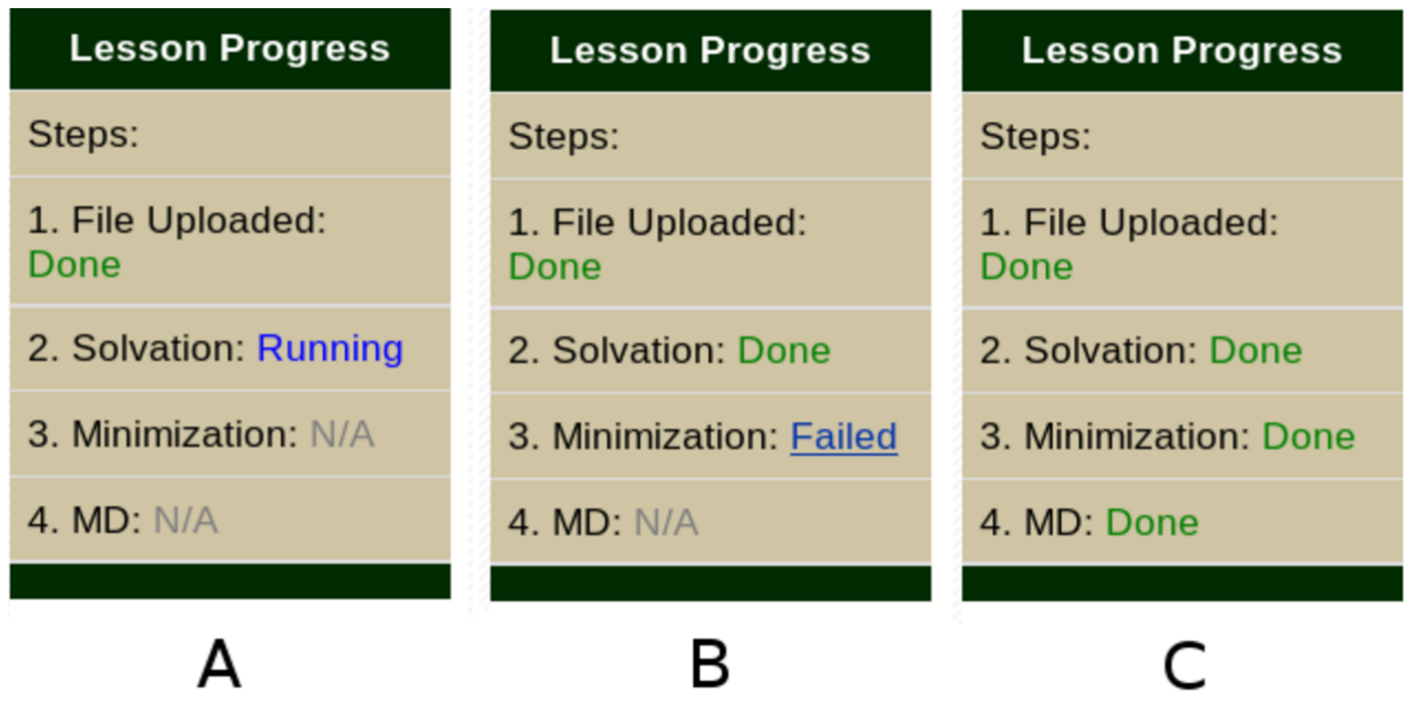
The lesson status bar of CHARMMing, which is displayed below the standard status bar. Panel A shows a lesson with solvation running, panel B shows an error condition, and panel C shows the status bar when the lesson is complete.

A lesson in CHARMMing is a list of tasks that a user must perform, beginning with creating a structure. Each lesson is independent and has an HTML page within CHARMMing that updates the user's progress as they complete tasks. Initially, the page for a particular lesson will contain instructions for submitting the structure that is to be used. Once each step is done, new text is added to the lesson page describing the next step that is to be performed. Once all steps are completed, the user is informed that the lesson is over. If a task is performed incorrectly at any time, an error message is displayed on the lesson page, and the step must be repeated until it is done correctly. All of the lesson pages are accessed under the “Lessons” subheader of CHARMMing's main menu. A drop down box was also added to the **Submit Structure** page that lets the user select with which, if any, lesson the structure is associated (see Figure 1). This allows users to start multiple lessons and even multiple copies of the same lesson. When a lesson is started, an additional status window is shown on the left-hand sidebar following the user's progress (Figure 2).

On structure upload and submission of a calculation (e.g., minimization or dynamics), CHARMMing checks to see if a lesson is in progress. If it is, then it calls a routine specific to that lesson to see if the calculation was set up correctly. Likewise, when a calculation is finished, CHARMMing sends its exit status and, depending on the type of calculation, results to another lesson-specific routine. Based on this information, the lesson logic can decide whether or not the submission or calculation was performed as intended. If it was sucessful, the user is allowed to move onto the next step of the lesson. If not, an error message is generated, which is displayed on the lesson HTML page, and the lesson status box also displays the fact that an error was detected.

## Lesson Implementation Details

### 

To demonstrate the capabilities of this infrastructure, six lessons have been implemented that together use a wide range of CHARMMing's functionality. These lessons are designed to be illustrative, since because of compute power limitations on the server and CHARMM license restrictions, all minimization and dynamics calculations are limited to 1,000 steps. Sites that have CHARMMing installed locally may lift this restriction for users covered under their CHARMM license. Also, users with access to CHARMM may download all files and scripts and continue calculations.

### Lesson One: Introduction

The objective of this lesson is to provide an introduction to the most basic features of CHARMMing. The student is instructed to create a periodic water box and run a short MD simulation. To accomplish this, the user uploads structure files describing a single water molecule and then uses the solvation functionality to create a rhombic dodecahedral unit cell. The user must then run minimization to place the structure in a low energy conformation. This step is often a prerequisite to further calculations, a facet that is described more completely in the tutorial. The behavior of the system is then studied via MD simulation. Since minimization puts the molecular system at a low energy state, it is necessary to “reheat” it to physiological temperature. Therefore, the user is instructed to start a heating simulation that increases the temperature from 210.15 K to 310.15 K over the course of 1,000 MD steps. For a publication-quality simulation, somewhat slower heating would likely be employed (e.g., 10,000–100,000 steps). The final outputs from the heating include a dynamics trajectory, and after completion the student should have a very basic understanding of the steps necessary to upload a simple structure into CHARMMing, solvate it, and perform a MD simulation with periodic boundary conditions (PBC).

### Lesson Two: Simulating Proteins

The major objective of lesson two is the application of simulation techniques to a protein system, the oxysterol binding protein complexed with cholesterol, which was previously studied by Singh et al. [17]. As part of this, additional details about force field parameters are introduced. The user is given a special structure file adapted from the protein data bank (www.pdb.org) [18], code 1ZHY [19], to upload into CHARMMing. CHARMM topology and parameter files containing cholesterol are provided [20], and the user must upload these into CHARMMing. Solvation is used, as in lesson one, but in this case the structure contains a net charge, so neutralization is introduced. This procedure adds counter-ions to the solvent to make the unit cell electrically neutral; important for the particle-mesh Ewald (PME) electrostatic method [21], [22]. Minimization is used as above to prepare the structure for dynamics. For this lesson, both heating and constant temperature (using the NVT ensemble, where number of particles [N], volume [V], and temperature [T] are held constant) dynamics are run, and the user is instructed to “make a movie” from the NVT trajectory. At the conclusion of this lesson, the user should have a better understanding of the techniques introduced in lesson one and be able to apply them to simple protein systems; furthermore, they should be able to use custom parameter files and know how to neutralize a charged system.

### Lesson Three: Enhanced Sampling/Self-Guided Langevin Dynamics (SGLD)

The objective of this lesson is to illustrate how molecular simulations can be used to study protein structure and conformation. For example, a critical component of current research efforts is determining how proteins fold [23]. It is intractable to conduct a complete conformational search or sampling study within the computational limits imposed by CHARMMing, and in fact, folding any more than relatively small (70–100 residue) polypeptides is currently beyond the capabilities of even the largest supercomputers; [24]–[26] however, students can still learn some basic principles. In this lesson, the user gives CHARMMing an amino acid sequence, GNNQQNY, which is found in the yeast protein Sup35 and has been crystallized (PDB: 1YJP) [27], and then instructs the software to generate an initial conformation. After minimization, a very short simulation (1 picosecond) is run utilizing an advanced sampling method, (SGLD) [28], which is designed to speed up slow, low frequency motion. The final step presents a simple analysis technique using the root mean squared differences (RMSD) between the starting and ending structures to give a measure of conformational change. Upon finishing this lesson, students should have a basic familiarity with the idea of methods that enhance conformational sampling in proteins. Furthermore, they should know how to simulate arbitrary amino acid sequences using CHARMMing.

### Lesson Four: Custom Residue Topology Files (RTF), Quantum Mechanics (QM)/Molecular Mechanics (MM)

So far, all of the lessons in CHARMMing have focused on classical simulation techniques using MM. In recent years, several techniques have been developed to combine MM with more accurate but computationally expensive QM methods. This has led to a hybrid class of methods, i.e., QM/MM [29]–[32], which are important for simulating a wide variety of systems, such as those undergoing reactions where a covalent bond is formed or broken [33]–[36]. The primary objective of CHARMMing's fourth lesson is to introduce QM/MM techniques; a secondary objective is to teach users about CHARMM's RTF format. The RTF describes the chemical topology, but not the interaction parameters, of various subunits (“residues”) such as amino acids, nucleic acids, and small molecules. In CHARMMing, several mechanisms exist for automatically generating a RTF and the associated parameters for many different types of biomolecules using the CHARMM General Force Field (CGenFF) [37]–[39].

To begin this lesson, the user creates a custom RTF for butane and then uploads it with the provided PDB file. Once the structure is uploaded, the user is instructed to calculate its single point classical energy to ensure that the custom RTF was made correctly. Afterwards, the user must calculate the energy again, but this time using QM/MM. Various schemes for managing the interactions between the classical and quantum regions have been devised [34]; however, for this lesson CHARMMing utilizes the additive QM/MM scheme implemented via the Q-Chem/CHARMM interface [40], [41]. The user is asked to use the Hartree-Fock method for the QM region (i.e., no correlation) in conjunction with the STO-3G basis set; the QM charge should be set to zero. The QM region is defined as half of the butane (i.e., one methyl and methylene group and its associated link atom). The final step in this lesson is to perform an energy minimization using QM/MM. At the conclusion of this lesson, the student should be able to perform simple QM/MM calculations in CHARMMing and have gained some insight into how to create and edit topology files for small molecules.

### Lesson Five: Coarse Grained Modeling

As mentioned previously, one of the major challenges facing the field of molecular modeling is elucidating large scale conformational changes in proteins. In the current lesson, coarse grained (CG) models [42]–[44], where multiple atoms are combined into a single interaction center (“bead”), are illustrated as an effective strategy to overcome many computational limitations. Full details of this are given in part II of this series of manuscripts.

### Lesson Six: Oxidation/Reduction Calculations

Oxidation and reduction reactions, where one atom involved in the reaction donates an electron to another atom, are an important part of many different biological reactions, such as those found in glycolysis or the Krebs cycle. Recently, CHARMMing has been extended to use the Adaptive Poisson-Boltzmann Solver (APBS) [45] for calculating reduction potentials of iron–sulphur metalloenzymes [46]. To illustrate the utility of these calculations, a lesson has been developed and integrated with new graphical capabilities and generic amino acid mutation functionality. Full details of this of this lesson and reduction potential calculations in CHARMMing are presented in Part III of this series of manuscripts.

### New Lessons

CHARMMing's lesson system is designed to allow the easy addition of new lessons, implemented as Django applications (see [4] for further information). Currently, this requires some knowledge of Python; however, we have developed an external Python script that creates the basic lesson infrastructure. From there, additional customization is still required; specifically, the lesson logic needs to be implemented and the user-facing instructions written. In the future, lessons will be created based on a “recorded” set of actions. For example, if the user uploads a stucture, performs a minimization, and identifies the energy; a lesson will then be created based on those actions and results.

## The CHARMM/CHARMMing Tutorial

### 

Although CHARMMing provides a simple, graphical introduction to molecular modeling with CHARMM, suitable for students at many levels, it is obvious that no such interface can fully meet the needs of more advanced users. However, too frequently, adequate resources do not exist to help new practitioners become comfortable with the technical details of available software. This is, sadly, especially true of the CHARMM program, which has comprehensive documentation but little practical advice on how to use the myriad of features. In practice, students tend to learn from more experienced members of their research group. In order to codify some of this “inherited wisdom,” we have developed a general CHARMM tutorial; available in the public domain at www.charmmtutorial.org. Additionally, each lesson links to this tutorial; for example, lesson one links to the tutorial's minimization page when describing why minimization is used.

The tutorial is organized roughly along the same lines as CHARMMing itself; however, being designed for more advanced users, it goes into significantly more depth than the simple introductory information given in the CHARMMing lessons. For example, the tutorial includes copious information about the CHARMM command scripting language (e.g., atom selections, constraints/restraints, variables, and loops) and numerous examples. The presentation of the command language is at the advanced level, describing variables, looping, and complex atom selection techniques, among other topics.

The tutorial also goes into some depth regarding the physics behind the calculations performed by CHARMM as well as the biological rationale for performing them. The importance of solute–solvent interactions, for example, is described on the Solvation page of the tutorial. Likewise, the pros and cons of various temperature controls and how they may be used in simulation is discussed. The placement of counter-ions around a solvated structure can have a dramatic effect on its behavior, and this topic, too, is considered.

One of the primary topics discussed is CHARMM's potential energy function, which is vital to understanding various simulation techniques such as energy minimization and MD simulation. The various bonded and nonbonded terms of the potential energy are explained and their functional forms given, followed by discussion of how CHARMM handles PBC. The physical and mathematical properties of PME, an Ewald-sum–based method for accurately computing electrostatic interactions of periodic systems, are also described as are the various nonbond cutoff methods available in CHARMM [47]. Molecular simulations textbooks should be consulted for more information on these subjects, and the 2009 CHARMM paper [3] also provides a good reference. The sections on **Minimization**, **Solvation,** and **Neutralization** build on this material and are described independently, as they are steps for preparing periodic systems.

Two sections of the tutorial are devoted to running dynamics: **Molecular Dynamics** and **Langevin Dynamics**. This discussion is long because there are many options to CHARMM's DYNAmics command, which is used for both types of simulation. How to produce trajectory files from simulations is also explained. A separate section is devoted to the **Analysis** of these trajectories; one of CHARMM's major strengths.

The final section of the tutorial is a **Full Example**, which walks through the set-up and execution of a MD simulation of Crambin (PDB code 1CBN) [48]. The reader is given detailed instructions on how to read in a PDB file, minimize, solvate, run dynamics, and perform some simple analyses. The actions that are performed and their ordering are those which might be used by an experienced practitioner performing a simulation study on a protein. All input scripts used in this example are available for download.

An appendix to the tutorial describes the installation process for CHARMMing, which is facilitated by an installation script designed for Debian or Ubuntu Linux based systems. This script allows institutions with a CHARMM license to install their own server for local use and remove restrictions on the number of steps that may be performed during any particular calculation.

CHARMM users and developers are encouraged to contribute to the tutorial. Please contact the administrator of http://www.charmmtutorial.org (listed on the front page of the website) if interested.

## Enhanced Visualization Features

### 

Recently, two new graphical visualization engines have been integrated into CHARMMing; JSmol [49] and GLmol [50]. JSmol is a fully functional JavaScript (JS) implementation of the Java-based Jmol [51] software. One major advantage is realized upon conversion to JS-based visualization: compatiblity with mobile computing platforms (e.g., Android, iOS) that do not support Java. Furthermore, JS-based user interfaces provide greatly enhanced web security as compared to Java-based technologies [52].

**Figure 3:**
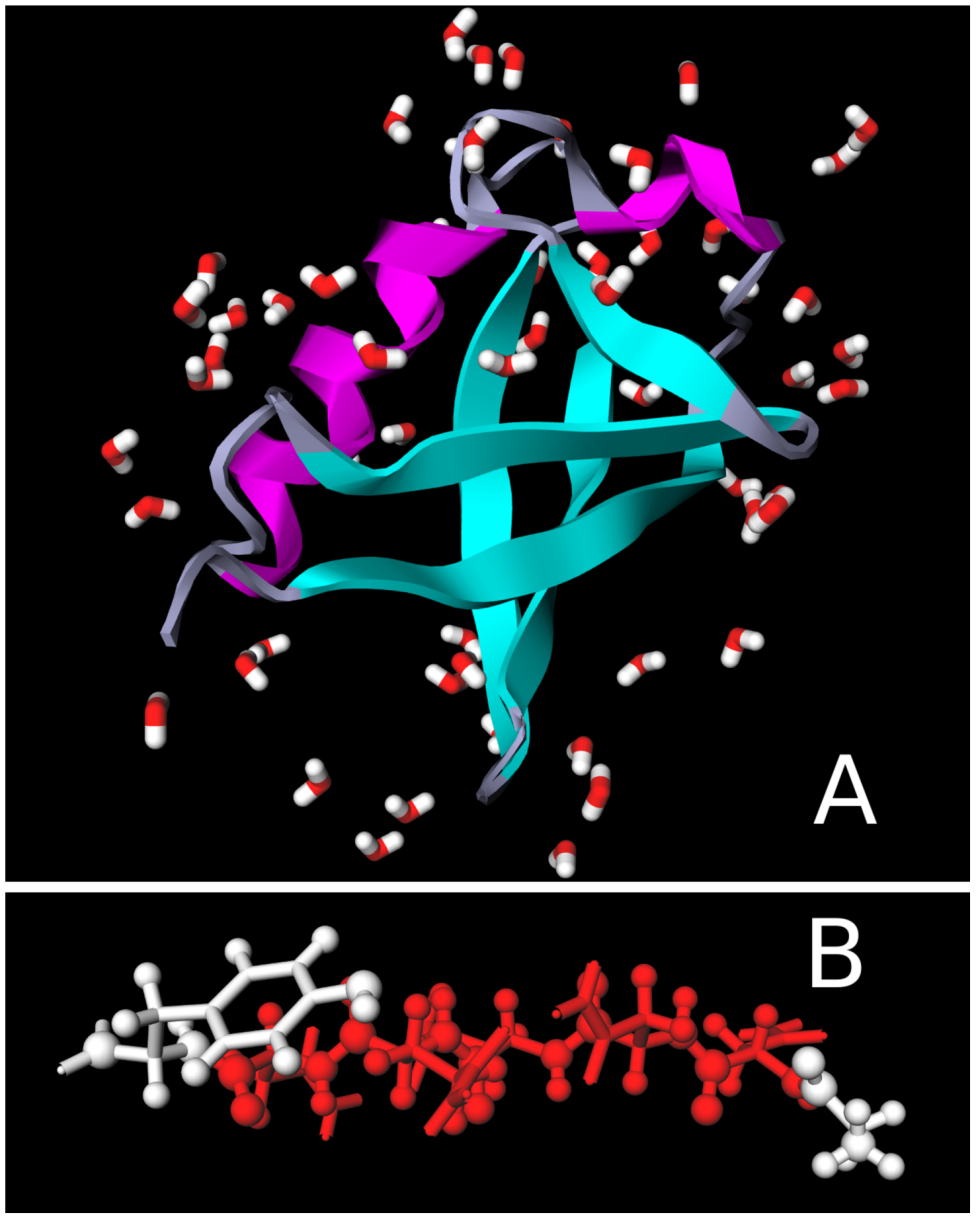
Examples of using GLmol for visualization. (A) Ubiquitin (PDB code 1UBQ [57]) displayed as ribbons with crystal waters. (B) A ball-and-stick representation of a small peptide (PDB code 1YJP [27]).

Although JSmol is extremely functional with wide-ranging compatibility, its graphical performance lags behind best-in-class desktop applications [53],[54]. Therefore, we also integrated GLmol into CHARMMing. GLmol is a 3-D molecular viewer based on modern web graphics; i.e., WebGL (Web Graphics Library). The WebGL-based GLmol offers advanced Graphics Processing Unit (GPU) accelerated visualization of 3-D molecular structures. In fact, performance and image quality (Figure 3) of GLmol can rival desktop applications; e.g., VMD [53], PyMol [54]. Although the quality and performance of GLmol is outstanding, JSmol offers far more functionality; therefore, we have chosen to use the latter to implement more recent CHARMMing features that require the end user to interact with a display. However, both GLmol and JSmol may be used for visualization.

## Conclusion and Outlook

### 

Knowledge of molecular simulation is an advanced topic that is becoming essential as a complement to experiment [55]. The aim of the CHARMMing project is to provide an easy to use interface to a powerful simulation package (CHARMM), so that those new to simulation may learn the underlying concepts and application without getting lost in the details of complex workflows and syntax. This aim is furthered by the development of interactive lessons, which may be used to teach these concepts and workflows in a more structured manner; making this an ideal tool for enhancing computational pedagogy at institutions that lack computational facilities. Furthermore, improved visualization capabilities will greatly facilitate the set-up and analysis of complex calculations and will be the basis for many new lessons in the future.

Despite the presence of powerful, web/graphical interfaces such as CHARMMing, ultimately it is necessary for those who wish to do advanced computational research to have detailed knowledge of the software they use. CHARMMing provides the input and output files for each calculation it performs, which are key to developing this deeper understanding. Furthermore, the tutorial highlighted in this manuscript is essential to bridging the gap between conceptual and technical understanding.

Finally, there has been interest in the use of CHARMMing as a general education tool. From personal communication, we know of at least four instances where CHARMMing was used in formal molecular simulation classes or tutorials. Several of the authors have been involved with one of these: the use of CHARMMing in a molecular simulation class at Georgetown University (CHEM-573 Computational Methods for Biological Macromolecules) [56]. It is anticipated that the developments described herein will assist in making molecular simulation techniques more accessible to students and other newcomers. CHARMMing is well placed as both a user and development platform to aid in these endeavors.
